# Supplementary material for: Validation of a pre-established triage protocol for critically ill patients in a COVID-19 outbreak under resource scarcity: A retrospective multicenter cohort study
Source: PLoS One. 2023 May 11;18(5):e0285690. doi: 10.1371/journal.pone.0285690 (PMC10174588; doi:10.1371/journal.pone.0285690)
Supplement: S1 Appendix — Detailed description of how a simulated triage cohort was generated and used to estimate the number of supplementary lives potentially saved by triage as compared with absence of triage, in a situation of saturated critical care capacities in a COVID-19 overwhelming outbreak. (PDF) [file pone.0285690.s001.pdf]

Simulated triage and resulting mortality and survival

The following approach was used to check whether, in a situation of saturated critical care capacities, the proposed triage strategy could save more lives as compared with no triage, and to quantify by which magnitude.

1	***The observed cohort served as a reference*** since no systematized triage strategy was used in the three participating centers during the study period. This was in line with the general situation in France at the time of the first epidemic wave of COVID-19, where ICU admission and treatment withholding or withdrawal decisions were at best made according to available non systematized ethical guidelines (1–5).
2	***The simulated triage cohort*** was derived from the reference cohort based on retrospectively allocated triage priority levels for initiation (day 0) or continuation (day 7 to 10, using actual recorded triage day for each patient) of critical care, following the SFAR/SSA triage protocol in situations of saturation (6). The following corrections of outcome and ICU length of stay (LOS) were applied.<br>• At day 0, patients triaged P4 would not have been admitted to ICU. Their LOS was thus set to 0. It was further assumed that they would all have ultimately died without critical care. Their outcome was thus set to death. Similarly, P3 patients would not have been admitted to ICU (unless later deteriorating and then newly triaged P1 or P2). Their LOS was thus also set to 0.<br>• At day 7 to 10, following the second triage step for patients who were still in ICU among those initially triaged P1 or P2 at day 0, treatment would have been withdrawn for P4 patients. This would have resulted in their transfer to palliative care or rapid ICU death. Similarly, P3 patients would have been transferred to post-ICU. Consequently, the LOS was set to end on triage day in both situations. It was also assumed that all P4 patients would have ultimately died after treatment withdrawal. Their outcome was thus set to death.
3	***The direct consequences of triage*** in terms of number of supplementary deaths and critical care resources (number of ICU patient days) made available were estimated through direct comparison of mortality and cumulated ICU LOS between the reference cohort and the simulated triage cohort.
4	***The number of supplementary patients potentially treated*** as a result of triage was then estimated. Under both hypotheses of lastingly saturated critical care capacities and continuous application of the triage strategy over the study period, we assumed that all critical care resources made available by triage would be used to treat other patients with higher priority.<br>• To that effect, we calculated the mean overall ICU LOS and the overall mortality of all patients initially triaged P1 or P2 at day 0 in the simulated triage cohort, thus with corrected LOS and outcome for further triage at day 7 to 10 when applicable.<br>• The supplementary number of P1 and P2 patients who could have received critical care thanks to triage was estimated by dividing the number of ICU patient days made available (*step 3*) by the mean ICU LOS of P1 and P2 patients under two-step triage.<br>• Their survival was estimated according to the survival rate of all P1 and P2 patients under two-step triage. Assuming that, without triage, all of them would have been denied critical care for lack of available resources and thus would have ultimately died, this survival estimated the supplementary number of patients who would have survived thanks to triage.
5	***The neat estimated number of lives potentially saved by triage*** under lastingly saturated critical care resources, as compared with no triage, was finally calculated by subtracting supplementary P4 deaths (*step 3*) from supplementary P1 and P2 survivors yielded (*step 4*).

A non pre-specified supplementary analysis was also performed following a similar method, but under the more severe assumption of such an overwhelming of critical care capacities that critical care withholding or withdrawal would not only be necessary in P4 patients, but even in P2 patients, in order to initiate or continue critical care only in P1 patients.

References

1. Emanuel EJ, Persad G, Upshur R, Thome B, Parker M, Glickman A, et al. Fair Allocation of Scarce Medical Resources in the Time of Covid-19. N Engl J Med. 2020 May 21;382(21):2049–55.

2. Rosenbaum L. Facing Covid-19 in Italy - Ethics, Logistics, and Therapeutics on the Epidemic's Front Line. N Engl J Med. 2020 May 14;382(20):1873–5.

3. Truog RD, Mitchell C, Daley GQ. The Toughest Triage - Allocating Ventilators in a Pandemic. N Engl J Med. 2020 May 21;382(21):1973–5.

4. White DB, Lo B. A Framework for Rationing Ventilators and Critical Care Beds During the COVID-19 Pandemic. JAMA. 2020 May 12;323(18):1773–4.

5. Comité consultatif national d'éthique. Enjeux éthiques face à une pandémie [Internet]. 2020 [cited 2020 Mar 31]. Available from: https://www.ccne-ethique.fr/sites/default/files/reponse_ccne_-_covid-19_def.pdf

6. Leclerc T, Donat N, Donat A, Pasquier P, Libert N, Schaeffer E, et al. Prioritisation of ICU treatments for critically ill patients in a COVID-19 pandemic with scarce resources. Anaesth Crit Care Pain Med. 2020;39(3):333–9.
