# Supplementary material for: Validation of a pre-established triage protocol for critically ill patients in a COVID-19 outbreak under resource scarcity: A retrospective multicenter cohort study
Source: PLoS One. 2023 May 11;18(5):e0285690. doi: 10.1371/journal.pone.0285690 (PMC10174588; doi:10.1371/journal.pone.0285690)
Supplement: S1 Table — Patient severity and outcome according to the priority level assigned at ICU admission (day 0, first step of the SFAR/SSA critical care triage protocol) in a situation of tension in critical care capacities. (PDF) [file pone.0285690.s002.pdf]

	P1 (N=158)	P2 (N=34)	P3 (N=17)	P4 (N=16)	Total (N=225)	p value
**Age (years)**	62.7 (11.8)	65.1 (9.3)	59.9 (12.6)	66.5 (16.0)	63.1 (11.9)	0.293[1]
**Gender**						0.714[2]
F	42 (27%)	7 (21%)	6 (35%)	4 (25%)	59 (26%)	
M	116 (73%)	27 (79%)	11 (65%)	12 (75%)	166 (74%)	
**BMI**						0.634[1]
N missing	2	1	0	0	3	
Mean (SD)	29.6 (6.8)	29.6 (4.6)	28.2 (6.1)	31.2 (8.7)	29.6 (6.6)	
**Hypertension**	90 (57%)	21 (62%)	3 (18%)	11 (69%)	125 (56%)	0.008[2]
**Type 2 diabetes**	47 (30%)	9 (26%)	2 (12%)	8 (50%)	66 (29%)	0.121[2]
**SAPS2**	37 [28 - 47]	50 [42 - 62]	24 [18 - 31]	49 [32 - 65]	40 [29 - 49]	< 0.001[3]
**Onset to ICU time (days)**						< 0.001[3]
N missing	4	2	0	0	6	
Median [Q1 - Q3]	8 [6 - 11]	7 [5 - 11]	7 [5 - 12]	4 [3 - 5]	7 [5 - 11]	
**SOFA score (day 0)**	5 [3 - 6]	9 [8 - 10]	2 [2 - 3]	7 [4 - 9]	5 [3 - 7]	
**Mechanical ventilation**	130 (82%)	34 (100%)	0 (0%)	12 (75%)	176 (78%)	
**PaO2/FiO2**						
N missing	2	0	0	0	2	
Median [Q1 - Q3]	124 [94 - 160]	108 [82 - 157]	272 [222 - 300]	118 [95 - 158]	125 [94 - 178]	
**Vasopressors**						
No	113 (72%)	1 (3%)	17 (100%)	8 (50%)	139 (62%)	
Dobutamine only	1 (1%)	0 (0%)	0 (0%)	0 (0%)	1 (0%)	
Epi-/Norepinephrine < 0.1 µg/kg/min	19 (12%)	3 (9%)	0 (0%)	3 (19%)	25 (11%)	
Epi-/Norepinephrine > 0.1 µg/kg/min	25 (16%)	30 (88%)	0 (0%)	5 (31%)	60 (27%)	
**Glasgow coma score**						
N missing	1	0	0	0	1	
Median [Q1 - Q3]	15 [15 - 15]	15 [15 - 15]	15 [15 - 15]	15 [14 - 15]	15 [15 - 15]	
**Serum creatinine (µmol/L)**						
N missing	1	0	0	0	1	
Median [Q1 - Q3]	72 [60 - 91]	115 [84 - 156]	76 [64 - 93]	105 [61 - 168]	77 [61 - 100]	
**Platelet count (10^9/L)**						
N missing	1	0	0	0	1	
Median [Q1 - Q3]	223 [181 - 290]	169 [126 - 262]	250 [190 - 293]	190 [130 - 261]	215 [167 - 289]	
**Bilirubin (µmol/L)**						
N missing	9	1	1	0	11	
Median [Q1 - Q3]	9.0 [7.0 - 14.0]	11.0 [8.0 - 14.0]	8.5 [6.0 - 12.0]	9.0 [6.0 - 12.0]	10.0 [7.0 - 13.8]	
**ECMO (day 0)**						
No	158 (100%)	33 (97%)	17 (100%)	16 (100%)	224 (100%)	
VV	0 (0%)	1 (3%)	0 (0%)	0 (0%)	1 (0%)	
VA	0 (0%)	0 (0%)	0 (0%)	0 (0%)	0 (0%)	
**Mortality**	39 (25%)	14 (41%)	0 (0%)	8 (50%)	61 (27%)	0.001[2]
**Discharged alive**	101 (64%)	12 (35%)	17 (100%)	8 (50%)	138 (61%)	< 0.001[2]
**Raw length of ICU stay (days)**	11 [5 - 21]	16 [8 - 32]	3 [2 - 12]	8 [3 - 16]	11 [5 - 21]	< 0.001[3]
**Length of ventilation (days)**	9 [4 - 17]	16 [6 - 28]	0 [0 - 7]	6 [1 - 15]	9 [4 - 17]	< 0.001[3]
**Treatment withheld or withdrawn**	27 (17%)	7 (21%)	0 (0%)	6 (38%)	40 (18%)	0.032[2]

1. Linear Model ANOVA
2. Fisher’s Exact Test for Count Data
3. Kruskal-Wallis rank sum test
