# Supplementary material for: Validation of a pre-established triage protocol for critically ill patients in a COVID-19 outbreak under resource scarcity: A retrospective multicenter cohort study
Source: PLoS One. 2023 May 11;18(5):e0285690. doi: 10.1371/journal.pone.0285690 (PMC10174588; doi:10.1371/journal.pone.0285690)
Supplement: S2 Table — Since no recovered cardiac arrest was recorded during initial ICU stay, the second step of priority allocation (on day 7 to 10) in tension was identical to that in saturation. (PDF) [file pone.0285690.s003.pdf]

	P1 (N=58)	P2 (N=13)	P3 (N=34)	P4 (N=46)	Total (N=151)	p value
**Priority level, day 0 (tension)**						0.026[1]
P1	38 (66%)	10 (77%)	29 (85%)	34 (74%)	111 (74%)	
P2	18 (31%)	1 (8%)	3 (9%)	5 (11%)	27 (18%)	
P3	0 (0%)	1 (8%)	0 (0%)	4 (9%)	5 (3%)	
P4	2 (3%)	1 (8%)	2 (6%)	3 (7%)	8 (5%)	

1. Pearson's Chi-squared test

Since no recovered cardiac arrest was recorded during initial ICU stay, the second step of priority allocation (on day 7 to 10) in tension was identical to that in saturation.
