# Supplementary figures and images for: Validation of a pre-established triage protocol for critically ill patients in a COVID-19 outbreak under resource scarcity: A retrospective multicenter cohort study
Source: PLoS One. 2023 May 11;18(5):e0285690. doi: 10.1371/journal.pone.0285690 (PMC10174588; doi:10.1371/journal.pone.0285690)

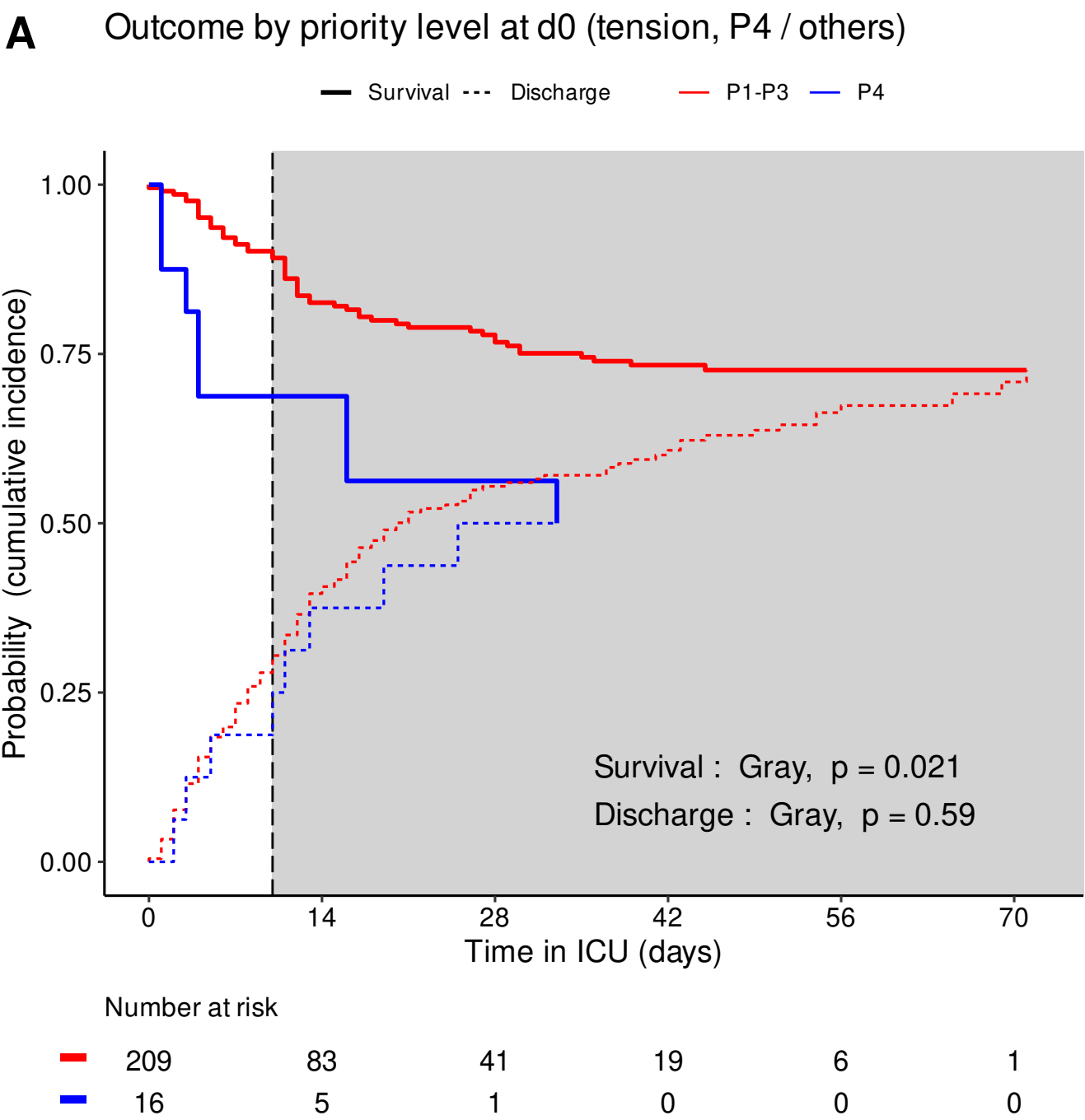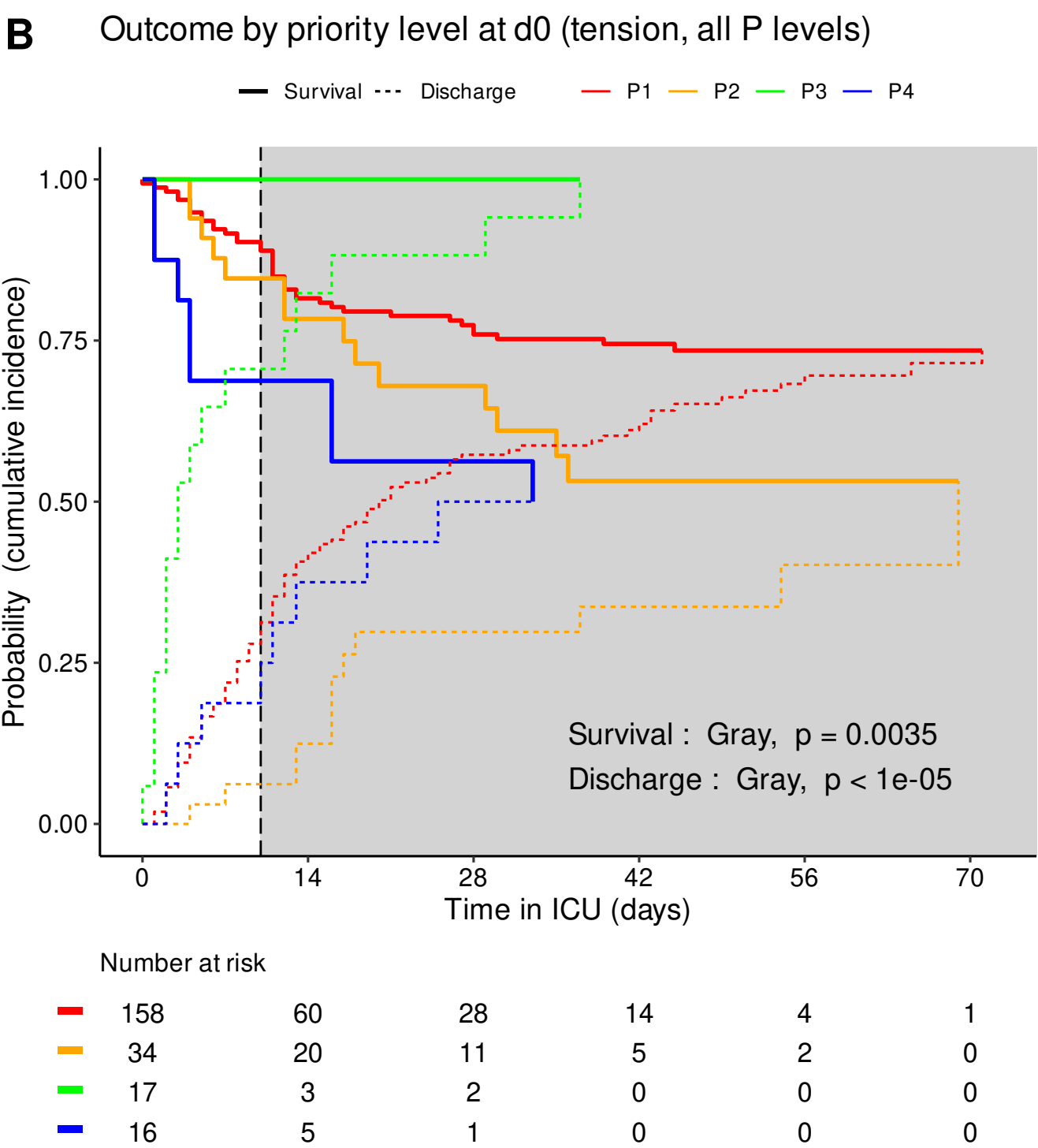

Supplement: S1 Fig — Cumulative incidence (c.i.) of alive discharge from ICU and survival (= 1 –c.i. of death in ICU) for COVID-19 patients. A: P4 compared with other priority levels at day 0. B: comparison between all priority levels at day 0. Shaded areas: initial prioritization no longer relevant due to reassessment. Since no recovered cardiac arrest was recorded during initial ICU stay, the second step of priority allocation (on day 7 to 10) in tension was identical to that in saturation. (PDF) [file pone.0285690.s004.pdf]
