# Supplementary material for: Validation of a pre-established triage protocol for critically ill patients in a COVID-19 outbreak under resource scarcity: A retrospective multicenter cohort study
Source: PLoS One. 2023 May 11;18(5):e0285690. doi: 10.1371/journal.pone.0285690 (PMC10174588; doi:10.1371/journal.pone.0285690)

Since no recovered cardiac arrest was recorded during initial ICU stay in the study cohort, the second step of priority allocation (re-assessment on day 7 to 10) for situations of tension yielded identical priority levels to that obtained for situations of saturation.

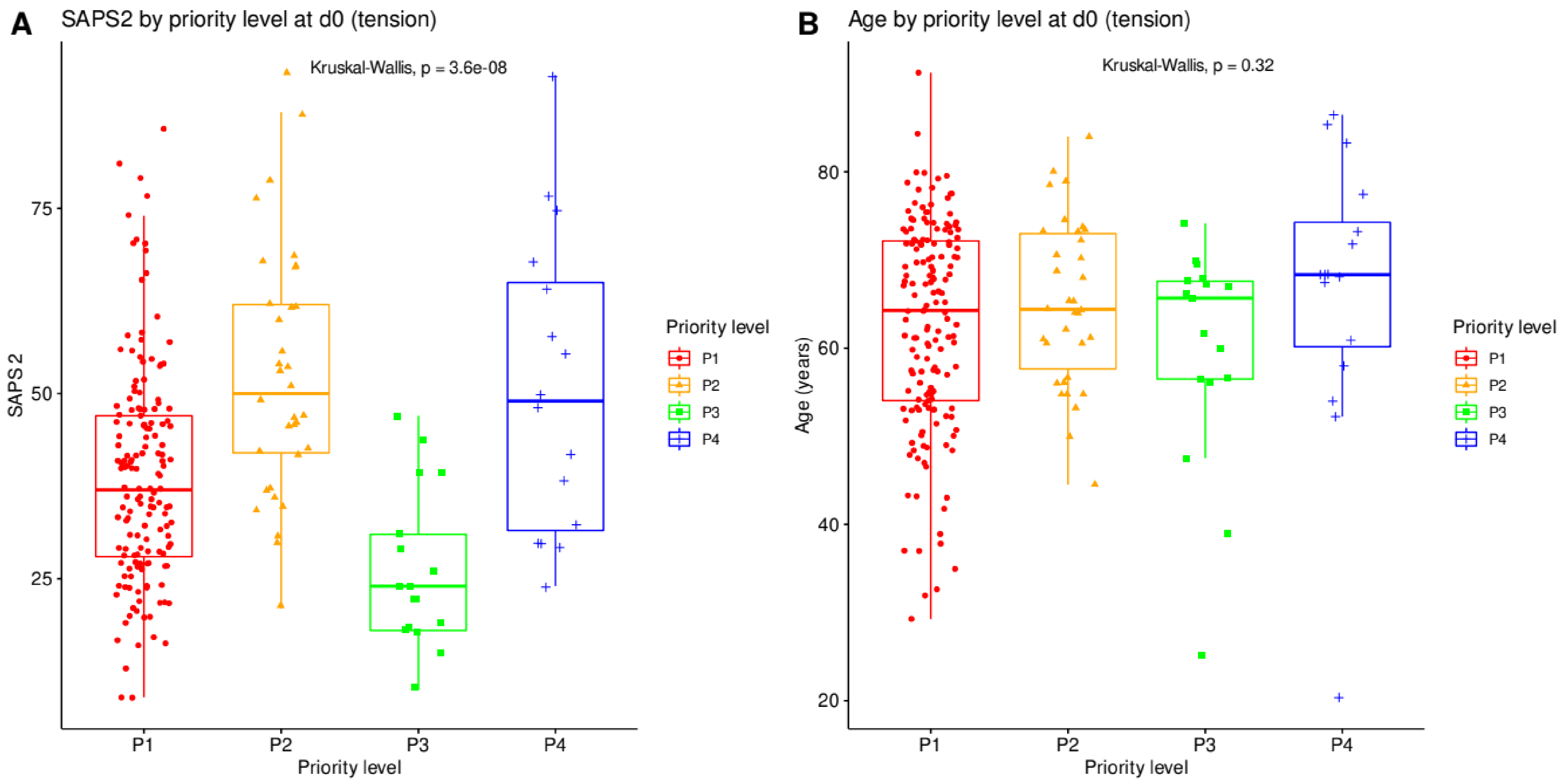

Supplement: S3 Fig — (PDF) [file pone.0285690.s006.pdf]
