# Supplementary material for: Validation of a pre-established triage protocol for critically ill patients in a COVID-19 outbreak under resource scarcity: A retrospective multicenter cohort study
Source: PLoS One. 2023 May 11;18(5):e0285690. doi: 10.1371/journal.pone.0285690 (PMC10174588; doi:10.1371/journal.pone.0285690)
Supplement: S1 File — (PDF) [file pone.0285690.s008.pdf]

ELSEVIER

Since January 2020 Elsevier has created a COVID-19 resource centre with free information in English and Mandarin on the novel coronavirus COVID-19. The COVID-19 resource centre is hosted on Elsevier Connect, the company's public news and information website.

Elsevier hereby grants permission to make all its COVID-19-related research that is available on the COVID-19 resource centre - including this research content - immediately available in PubMed Central and other publicly funded repositories, such as the WHO COVID database with rights for unrestricted research re-use and analyses in any form or by any means with acknowledgement of the original source. These permissions are granted for free by Elsevier for as long as the COVID-19 resource centre remains active.

Anaesth Crit Care Pain Med 39 (2020) 333–339

SFAR
Société Française d'Anesthésie et de Réanimation

Special article

Prioritisation of ICU treatments for critically ill patients in a COVID-19 pandemic with scarce resources[☆,☆☆]

Thomas Leclerc [a,b,*], Nicolas Donat [a], Alexis Donat [c], Pierre Pasquier [a,b], Nicolas Libert [a], Elodie Schaeffer [d], Erwan D'Aranda [e], Jean Cotte [e], Bruno Fontaine [d,b], Pierre-François Perrigault [f,g], Fabrice Michel [i,g], Laurent Muller [j,g], Eric Meaudre [e,b], Benoît Veber [h,g]

[a] *Percy military teaching hospital, Clamart, France*
[b] *Val-de-Grâce military medical academy, Paris, France*
[c] *Legouest military teaching hospital & Mercy regional hospital, Metz, France*
[d] *R. Picqué military teaching hospital, Bordeaux, France*
[e] *Sainte Anne military teaching hospital, Toulon, France*
[f] *Gui de Chauliac hospital & Montpellier University, Montpellier, France*
[g] *Ethics committee, French society of anaesthesia and critical care (SFAR), Paris, France*
[h] *Charles Nicolle hospital & Rouen Normandie University, Rouen, France*
[i] *APHM, Aix Marseille Univ, UMR ADES n°7268, EFS, CNRS, Marseille, France*
[j] *CHU Nîmes Caremeau, Nîmes, France*

ARTICLE INFO

Article history:
Available online 17 May 2020

Keywords:
COVID-19
Pandemic
Critical care
Ethics
Prioritisation
Triage

ABSTRACT

Background: Relying on capacity increases and patient transfers to deal with the huge and continuous inflow of COVID-19 critically ill patients is a strategy limited by finite human and logistical resources.
Rationale: Prioritising both critical care initiation and continuation is paramount to save the greatest number of lives. It enables to allocate scarce resources in priority to those with the highest probability of benefiting from them. It is fully ethical provided it relies on objective and widely shared criteria, thus preventing arbitrary decisions and guaranteeing equity. Prioritisation seeks to fairly allocate treatments, maximise saved lives, gain indirect life benefits from prioritising exposed healthcare and similar workers, give priority to those most penalised as a last resort, and apply similar prioritisation schemes to all patients.
Prioritisation strategy: Prioritisation schemes and their criteria are adjusted to the level of resource scarcity: strain (level A) or saturation (level B). Prioritisation yields a four level priority for initiation or continuation of critical care: P1–high priority, P2–intermediate priority, P3–not needed, P4–not appropriate. Prioritisation schemes take into account the patient's wishes, clinical frailty, pre-existing chronic condition, along with severity and evolution of acute condition. Initial priority level must be reassessed, at least after 48 h once missing decision elements are available, at the typical turning point in the disease's natural history (ICU days 7 to 10 for COVID-19), and each time resource scarcity levels change. For treatments to be withheld or withdrawn, a collegial decision-making process and information of patient and/or next of kin are paramount.
Perspective: Prioritisation strategy is bound to evolve with new knowledge and with changes within the epidemiological situation.
© 2020 Société française d'anesthésie et de réanimation (Sfar). Published by Elsevier Masson SAS. All rights reserved.

1. Background

The rapid spread of the COVID-19 pandemic results in a huge continuous and prolonged inflow of critically ill patients with a very high incidence of ARDS [1]. Challenge is then to provide all patients with the best possible quality of care by allocating

☆ French Society of Anaesthesia and Critical Care (SFAR)–Ethics committee.
☆☆ French military medical service (SSA)–Val-de-Grâce military medical academy, chair of anaesthesia, critical care and emergency medicine.
* Corresponding author at: Head of Burn treatment centre, Percy military teaching hospital, 101, avenue Henri Barbusse, 92141 Clamart, France.
E-mail address: thomas.leclerc@m4x.org (T. Leclerc).

https://doi.org/10.1016/j.accpm.2020.05.008
2352-5568/© 2020 Société française d'anesthésie et de réanimation (Sfar). Published by Elsevier Masson SAS. All rights reserved.

available resources in order to maximise the probability of cure for the greatest number while abiding by ethical principles.

1.1. Initial strategy

Initial management strategy of such a crisis relies on expanding treatment capacities and on transferring patients from overwhelmed areas to comparatively spared ones. This strategy finds its limits:

- logistically, because of the scarcity or even the lack of equipment and supplies (such as ventilators, syringe pumps, drugs, personal protective equipment) or evacuation vectors;
- humanly, because of the limited number of appropriately trained healthcare professionals, when the rhythm of the epidemic overwhelms local, regional or national capacities.

Should those limits not properly be anticipated and taken into account, such a strategy would result in an unacceptably lowered overall management quality for all patients and an increase in mortality due to a saturation effect [2].

Indeed, in such a situation, the huge and continuous inflow of patients combined with their high length of ICU stay results in overwhelmed treatment capacities for several weeks at least [3], as compared with trauma disasters where the saturation effect is typically a few days long.

1.2. Strategy when saturation occurs

When the healthcare system reaches saturation, strategy must be adjusted to fairly allocate scarce resources, in order to maximise the benefit of the greatest number of patients. This prioritisation strategy is also called triage, as in war or disaster medicine. It is both individually and collectively ethical, provided it bans arbitrary decisions and relies on objective elements that are shared between healthcare professionals, decision-makers and the whole community.

Prioritisation does not conflict with ethical principles regarding ICU access in the situation of a COVID-19 epidemic [4–9]. It expands them into an operational framework that can be used by critical care providers, emergency physicians and intensivists, so that they can make quick and coherent decisions that abide by those principles.

This paper sets out the rationale for prioritisation and proposes a prioritisation strategy that can be used when ICU resources are scarce during a COVID-19 epidemic. If critical care providers widely adopt and share this strategy, it can guarantee treatment equity between treatment structures among affected areas.

2. Rationale

2.1. Scope

The following prioritisation strategy deals with all critically ill patients, irrespective of their COVID-19 status (confirmed, suspected or COVID-free) for whom initiation or continuation of critical care treatments is being considered when ICU treatment capacities are scarce. Two main situations are identified, depending on the gap between available resources and identified needs for ICU treatment. These can be dealt at regional or national level.

- level A: strain. Though already increased, ICU remaining capacity is scarce. Transferring patients to less strained ICUs is also reaching its limits;
- level B: saturation. Despite capacity increase, ICUs are not able to handle more patients. Patient transfers to less strained ICUs are not sufficient to keep the situation under control.

Both situations should lead to adapting initial admission and continuation of critical care treatment criteria.

Assessing whether the situation falls into one of the aforementioned levels should be the responsibility of decision-makers at political or administrative levels and not that of attending physicians. In France, this would involve the General Health Directorate (DGS, national level), the Regional Health Agencies (ARS, regional level), or in extreme cases hospital directors.

2.2. Ethical principles

In all circumstances including resources scarcity, fundamental ethical principles must be complied with [5,8]:

- respecting human dignity, which translates into respecting autonomy, beneficence and non-maleficence, and which excludes any selection criteria based on "social utility";
- solidarity requirements;
- fairness, which can rely on distributive justice in such circumstances.

Thus, collecting as soon as possible patient's wishes, if need be through the next of kin, remains paramount. It is all the more important in saturation situations: it would clearly be unethical to allocate scarce resources to a patient for a treatment which he or she does not wish to receive.

When human or material resources do not allow for all patients to receive the treatments which would ideally be necessary for each of them, these principles unfold into the following objectives [10,11]. They should be transparently stated.

2.2.1. Ensuring equitable access to treatments

Scarce resources need to be allocated without discrimination on grounds of age, sex, nationality, geographical origin, social status, economic situation or disability. None of these criteria by themselves can justify different allocation of resources. This does not preclude some of them from being considered within general assessment of prognosis. This may typically be the case of age or especially chronic respiratory impairment.

Resource allocation process must be fair, based on objective criteria, and transparent, in order to avoid arbitrary decisions.

2.2.2. Maximising the benefits of scarce resources

When needs exceed resources, given the lethality of COVID-19 and the magnitude of the inflow of patients, main challenge is to minimise the number of deaths, and secondarily to maximise the number of preserved life years. Thus, every decision must aim at maximising chances of survival not only for each individual patient, but foremost of as many patients as possible. This leads to prioritising treatments for patients who have the highest probability of benefiting from them.

2.2.3. Considering indirect benefits

Due to their caring for infected patients, healthcare professionals are exposed to a higher risk of being infected by SARS-CoV-2 and to a higher risk of physical exhaustion and psychological distress. Should they become unable to work in a context where baseline human resources are already insufficient or barely sufficient to meet healthcare requirements, this would result in even more deaths. Protecting exposed caregivers from these risks thus appears both as an ethical requirement in terms of distributive justice and as an operational requirement since it helps maximise the number of lives saved. Occupationally exposed healthcare professionals should be granted priority access to critical care treatments if need be. Indeed, the community can fairly expect that these professionals take risks,

even mitigated, for the greater good if it ensures that they will be offered the best chances if infected. This is not based on a supposedly higher social utility but merely on the objective of maximising the number of lives saved [10,12]. This also applies to healthcare professionals deemed high risk because of their age or comorbidities. Protecting them requires ensuring they are not exposed to SARS-CoV-2. If circumstances are such that they are exposed, then this should lead to ensuring that they have priority access to ICU treatments. However, once access has been granted, reassessments will be based on standard evolution criteria.

A similar approach can also be used for people exposed through their contribution to research on COVID-19 (researchers or subjects enrolled in clinical studies), or for people fulfilling critical missions to keep the outbreak under control.

2.2.4. Giving priority to the most penalised as a last resort

Such an approach is only considered when purely medical criteria are not sufficient to make a difference on anticipated outcome. It would lead, for instance, to prioritising the younger of two patients with identical severity and prognosis factors, since he or she would potentially loose more life years if not granted access to treatment [10]. This would also be coherent with the secondary objective of maximising the number of life years preserved. Such decisions are extremely difficult to make since they are not solely based on medical criteria. This guidance is therefore important for those having to make them.

2.2.5. Prioritising all patients, regardless of COVID-19 status

ICU resources scarcity does not only impact COVID-19 patients, even more so since non-COVID ICU resources are drastically reduced to meet ICU needs of COVID-19 patients. Applying an identical prioritisation scheme for all ICU patients, regardless of their COVID-19 status, is both an ethical requirement in terms of fairness and key to operational efficiency. However, given the contagiousness of SARS-CoV-2 and the necessity to limit its transmission, COVID and non-COVID patients will in most cases be cared for in separate units, each with its own dedicated resources. This means scarcity levels (i.e. level A/strain or level B/saturation) will need to be evaluated separately and prioritisation schemes applied accordingly.

2.3. Prioritisation criteria

2.3.1. Scalability

Prioritisation criteria rely on objective data. Iterative reassessment is required to take into account newly gained knowledge, all the more so in the case of COVID-19 where insights are rapidly changing.

Prioritisation criteria are adjusted to take into account the level of resource scarcity (level A/strain or level B/saturation).

2.3.2. Selected criteria

Short term prognosis is the key criterion when deciding to initiate or continue critical care treatments [11]. Highest priority is given to patients having the highest probability of benefiting from such treatments.

At admission, it is estimated through acute and chronic comorbidity criteria. Age is not by itself a criterion, but it is indirectly considered through its association with these comorbidities and through its impact on short-term life expectancy.

During ICU stay, it is estimated through iterative assessment of organ failures and their evolution trends (deterioration, stabilisation, improvement or response to treatment) in order to discuss treatment continuation, withholding or withdrawal.

2.3.3. Excluded criteria

Prioritisation on a “first come, first served” basis is not equitable. This would lead to favouring those patients which live closer to a healthcare facility. It would also penalise those patients who, having complied with stay-at-home and social distancing measures for the common good, become sick at a later stage. To avoid such a situation, treatments need to be iteratively reassessed: prioritisation regarding treatment continuation must be performed along with prioritisation regarding treatment initiation [13].

Prioritisation based on randomisation or so-called lottery, as put forward by some authors to allocated scarce resources between similar patients [10], would be unfair and would negate the very uniqueness of each and every life. No ethical prioritisation strategy can incorporate such an approach.

2.4. Additional elements

Cardiac arrest, except if due to a clearly identified reversible cause, should not lead to cardiopulmonary resuscitation attempts when resources are scarce.

Extracorporeal membrane oxygenation (ECMO) is usually not adequate in this context [14,15], except in specific expert centres for exceptional and duly justified situations [16].

3. Prioritisation tools

3.1. Priority levels

Four priority levels are set, from P1 to P4, with usual triage colour codes. Their meaning and consequences on management decisions are summarised in Table 1.

Table 1
Priority levels for allocating ICU treatments to critically ill patients in COVID-19 pandemic with scarce resources.

	Situation	Management decisions
P1	Patient will likely not survive without critical care treatments and has a high probability of benefiting from them.	Initiate critical care treatments or continue them without restriction.
P2	Patient will likely not survive without critical care treatments but an intermediate probability of benefiting from them.	Initiate critical care treatments or continue them.<br>In similar situations, low availability treatments (such as nitrogen monoxide, NO) should rather be allocated to P1 patients.
P3	Patient does not currently require critical care treatments (or not yet or no longer).<br>Those treatments should be allocated to patients who need them more, and thus have a higher probability to benefit from them.	Do not initiate critical care treatment, unless worsening condition prompts reassessment.<br>Discharge from ICU to appropriate downstream unit (respiratory weaning & rehabilitation, medical ward).
P4	Despite critical illness, which might lead to critical care treatments out of resource scarcity, probability of the patient benefiting from them is low.<br>It would be unwise to allocate scarce resources which might be missed by patients with a higher probability of benefiting from critical care treatments.	Do not initiate, withhold or withdraw critical care treatments as appropriate, in compliance with good practice and current regulations.<br>Provide at least optimal palliative care in any situation.

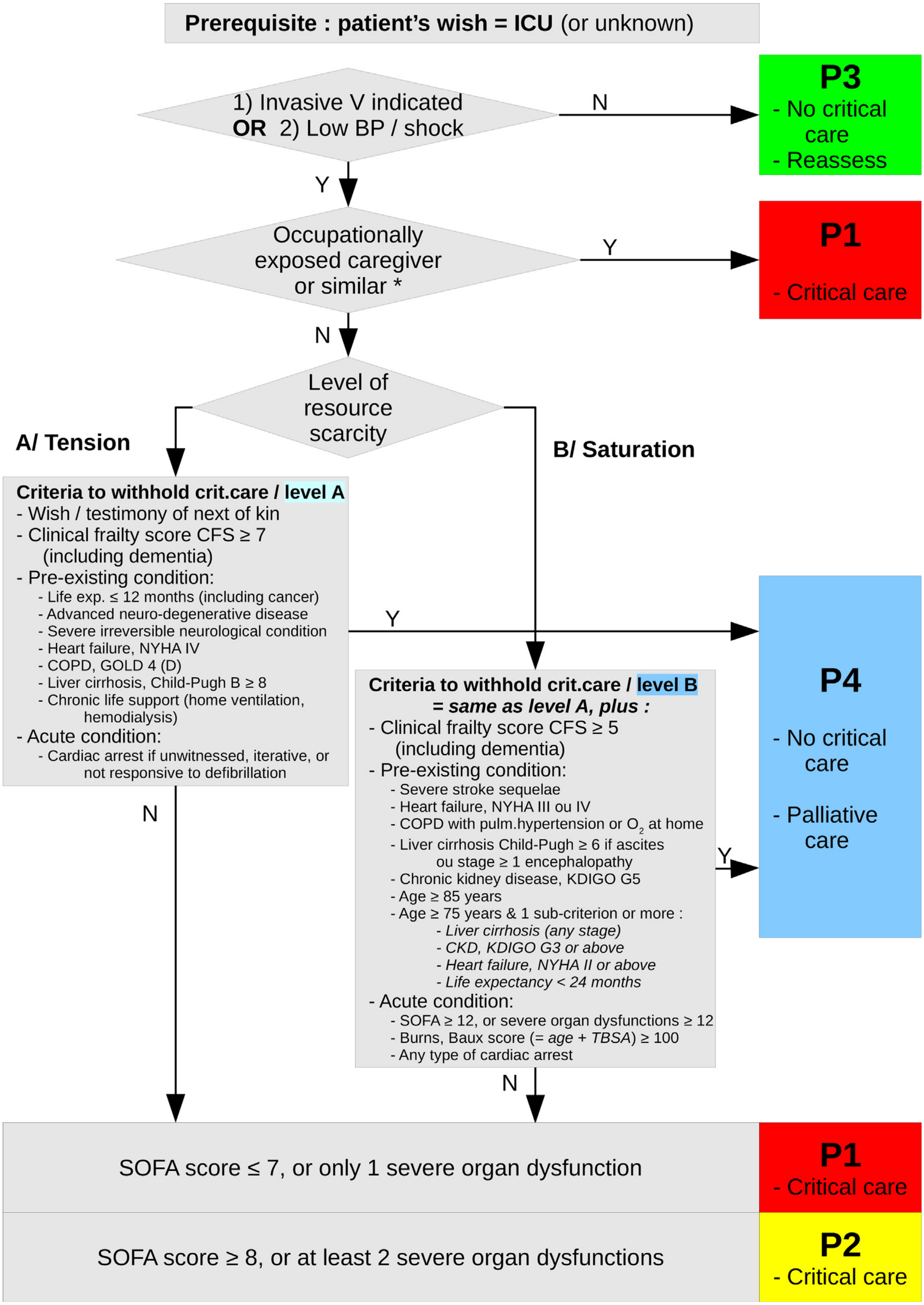

Fig. 1. Critically ill patients in COVID-19 pandemic with scarce resources: prioritisation for initiation of critical care treatments.

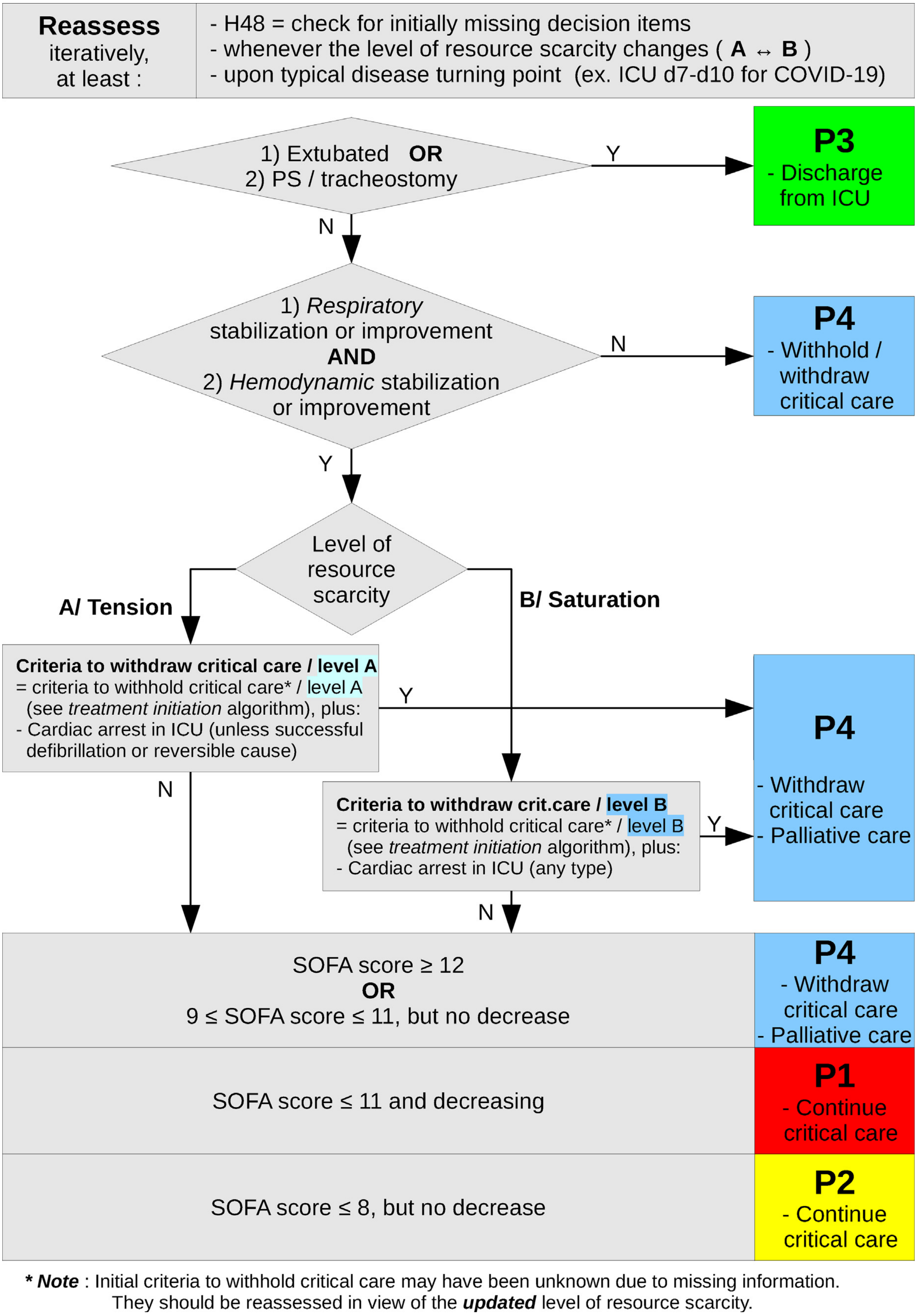

Fig. 2. Critically ill patients in COVID-19 pandemic with scarce resources: prioritisation for continuation of critical care treatments.

3.2. Implementation

Triage of patients in mass casualty situations is always a continuous, dynamic and evolutive process. This is all the more true in situations with a continuous and long lasting, saturating inflow of patients such as the COVID-19 pandemic. As previously mentioned (see 2.3.), prioritisation is iteratively reassessed.

- An initial priority level is set for the initiation of critical care treatments (Fig. 1). Whenever possible, every effort should be made to collect corresponding decision elements before the patient becomes critically ill. This allows anticipating the situation where a worsening condition would prompt discussion about withholding or withdrawing treatments including critical care. This is in line with good practice and legal requirements, including placing the patient's wish first, making collegial, individualised and substantiated decisions, informing patients and/or next of kin, and duly tracing the whole process.
- Priority level for the continuation of critical care treatments is iteratively reassessed whenever necessary (Fig. 2), and at least:
  - after 48 h to check for decision elements that might have been missing upon ICU admission (or when those elements become known);
  - when the level of resource scarcity changes (A or B), either improving or worsening;
  - upon the usual turning point of the disease, typically between d7 and d10 for COVID-19 [1,17].

P4 priority level results in a decision not to initiate critical care treatments, or to withhold or to withdraw them. Current guidelines [4,6–8] summarised in Appendix A are applicable to comply with corresponding good practice and current regulations. In order to ensure a collegial decision, a dedicated external team comprising ICU experts can support the decision-making process. This can help to alleviate the burden on physicians in charge, which can be especially strenuous as observed in Italian teams [13].

In exceptional situations, similarly to disaster medicine, the typical temporality of a treatment withholding or withdrawal process cannot always be complied with, especially for ICU admissions. In such exceptional cases, because herein described prioritisation schemes are widely shared beforehand by caregivers, a decision made in compliance with them by a physician compelled to decide without delay would be deemed implicitly collegial, the aim being that an explicit collegial confirmation be reached as early as possible.

As all triage tools, the present prioritisation schemes are no substitute to clinical judgement of physicians in charge. However, the higher the level of resource scarcity and the more homogenous the use of these prioritisation schemes within the healthcare system, the higher the benefit will be in terms of preserved lives.

3.3. Prioritisation schemes

Prioritisation schemes for initiation of critical care treatments and for their continuation are respectively given in Figs. 1 and 2.

They are widely based on following sources.

A Canadian triage tool was developed to prepare for a possible influenza pandemic [18]. It was further evaluated using data from a retrospective cohort of severe influenza cases [19]. The present prioritisation schemes have retained its four priority levels along with reassessments based on Sequential Organ Failure Assessment (SOFA) score [20] (see Appendix B) and its variations. Its concept of minimal qualification for survival was dropped in favour of reassessment at the usual turning point in natural history of disease. This seems better suited to current knowledge regarding COVID-19 [1,21].

The Swiss Academy of Medical Sciences issued specific triage recommendations for intensive care treatment under resource scarcity in COVID-19 pandemic [11]. The present prioritisation schemes have retained their distinction between two stages of resource scarcity, explained herein as level A–strain and level B–saturation.

They also integrate considerations from recent publications that specifically address COVID-19 [10,12,13], as synthesised in the rationale (see 2.). Among other elements, the proposition to give priority to exposed caregivers and similar workers in order to take into account indirect benefits (see 2.2.) comes from these sources.

Criteria to withhold critical care have been adapted with following key changes and precisions:

- Structuring criteria has been strengthened;
- In order to assess neurological dysfunction in sedated patients within SOFA score determination [20], the Glasgow coma scale (GCS) value to consider should be either the last GCS value measured before sedating the patient, or current GCS value with correction for verbal response (V) in a no longer sedated patient who still has an endotracheal tube or cannula;
- For patients with chronic kidney disease, only glomerular filtration rate (G criterion) of the KDIGO classification [22] (see Appendix C) is considered, as it can readily be obtained from a simple blood sample, contrary to albuminuria (A criterion);
- Severe traumas have been removed, considering the lack of sufficiently simple and robust tools to assess probability of benefiting from critical care for trauma patients beforehand;
- Baux score (= age in years + total body surface area burned in %) has been chosen as burn severity criterion due to its simplicity and excellent prognostic value [23], way better than burned surface area alone. Smoke inhalation is not considered, as its diagnosis is impossible without fiberoptic bronchoscopy;
- Clinical frailty score (CFS, see Appendix D) is used to assess clinical autonomy [24], in line with French recommendations regarding treatment withholding/withdrawal procedures [4,6,8]. Thresholds are proposed, based on tentative assessment of the probability that surviving patients could be successfully rehabilitated afterwards;
- Simplifications have been brought to work out partial redundancies.

Risk factors for severe COVID-19 are considered only if they are also already known to be correlated with increased ICU mortality. This leads to keeping diabetes, hypertension and obesity out of the process so far [17,25].

3.4. Evaluation and revision of prioritisation tools

Two pitfalls must be equally avoided in any situation of mass casualty or mass patient inflow, because both are associated with increased mortality.

Lack of prioritisation, or overtriage (giving high priority to too many patients), could not compensate for saturation of ICU capacities. It would lead to deprive patients, with high probability to benefit from critical care treatments, from receiving these treatments, as resources would already be mobilised for patients with worse prognosis.

Excess of prioritisation, or undertriage (giving high priority to not enough patients), would lead to unduly withholding critical care treatments for patients with high probability to benefit from them.

All criteria used in the current version of prioritisation schemes will need to be reconsidered following new knowledge regarding COVID-19 and changes in the epidemiological situation. This is especially true for thresholds: these might need adjusting to the

magnitude of saturation, just like it was necessary in Italy when overwhelming was maximal [26].

Other approaches have been proposed, such as assigning a priority score between 1 and 8 based on acute severity criteria and on pre-existing conditions with various correction factors. This daily reassessed score results in a three-level priority for patients in whom critical care would be indicated without resource scarcity [12]. This scheme was discarded here due to its complexity, but comparison between prioritisation tools should be considered to help improving them.

4. Conclusion

The COVID-19 pandemic challenges health systems with an inflow of critically ill patients of an unprecedented magnitude. Measures taken by decision-makers to minimise the gap between healthcare needs and resources rely on reducing viral transmission and on increasing treatment and patient transfer capacities. Considering the knowledge gap regarding SARS-CoV-2 and experience gained from the first affected regions, whether these measures can be sufficient to prevent healthcare facilities from being overwhelmed at some time point cannot be certain.

Preparing for situations where resources would be too scarce to allow treating all critically ill patients is thus a key responsibility, similarly to war and disaster medicine. In such cases, prioritisation is the only way to save the greatest number of lives. Advance thinking allows for a sound, ethical and shared prioritisation strategy. If the crisis develops into an overwhelming situation, relying on such a strategy avoids arbitrary decisions and undue deaths. If it does not, prioritisation simply does not need to be implemented. In all cases, pre-defined prioritisation schemes can be further improved by confronting them with real patient datasets when they become available, but waiting for that time to design these schemes would unduly cost lives if patient overflow occurs in-between. In that matter, anticipation is the first ethical requirement.

Authors' contributions

TL and ND had the initiative of this work and wrote the manuscript.

AD, PP, NL, ES, EA, JC, BF and EM revised the manuscript.

TL, BF and EM (head of chair) conducted its endorsement by the French military medical service–Val-de-Grâce military medical academy, chair of anaesthesia, critical care and emergency medicine.

PFP, FM, LM and BV (president of ethics committee) revised the manuscript and conducted its endorsement by the French Society of Anaesthesia and Critical Care (SFAR) and its ethics committee.

Disclosure of interest

The authors declare that they have no competing interest.

Appendix A. Supplementary data

Supplementary data associated with this article can be found, in the online version, at https://doi.org/10.1016/j.accpm.2020.05.008.

References

[1] Yang X, Yu Y, Xu J, Shu H, Xia J, Liu H, et al. Clinical course and outcomes of critically ill patients with SARS-CoV-2 pneumonia in Wuhan, China: a single-centered, retrospective, observational study. Lancet Respir Med 2020. http://dx.doi.org/10.1016/S2213-2600(20)30079-5.

[2] Ranney ML, Griffeth V, Jha AK. Critical Supply Shortages – The Need for Ventilators and Personal Protective Equipment during the Covid-19 Pandemic. N Engl J Med 2020. http://dx.doi.org/10.1056/NEJMp2006141 [NEJMp2006141].

[3] Grasselli G, Pesenti A, Cecconi M. Critical Care Utilization for the COVID-19 Outbreak in Lombardy, Italy: Early Experience and Forecast During an Emergency Response. JAMA 2020. http://dx.doi.org/10.1001/jama.2020.4031.

[4] Azoulay E, Beloucif S, Vivien B, Guidet B, Pateron D, Le Dorze M. [Decision to admit patients to Intensive care units in COVID-19 pandemic (in French)]. Ile-de-France: Regional recommendations - ARS; 2020 [https://www.srlf.org/wp-content/uploads/2020/03/ARS-ETHIQUE-COVID-final.pdf (accessed March 31, 2020)].

[5] Comité consultatif national d'éthique. Ethical considerations in pandemic (in French); 2020 [https://www.ccne-ethique.fr/sites/default/files/reponse_ccne_-_covid-19_def.pdf (accessed March 31, 2020)].

[6] Comité d'éthique de la S.F.A.R.. About medical decisions regarding admission of patients to critical care units during pandemic (in French); 2020 [https://sfar.org/download/comite-ethique-a-propos-des-decisions-medicales-dad-mission-des-patients-en-unite-de-soins-critiques-en-context-pandemique-quelques-reperes-ethiques/(accessed March 31, 2020)].

[7] Commission d'éthique de la S.R.L.F.. Admission criteria and treatment modalities in intensive care units in pandemic (in French); 2020 [https://www.srlf.org/wp-content/uploads/2020/04/20200410-Reco-Ethique-SRLF.pdf (accessed April 10, 2020)].

[8] de Montgolfier S, Lamblin A, Leport C. Operational multidisciplinary guideloines: ethical and strategic considerations regarding access to critical carein COVID-19 pandemic (in French); 2020 [https://www.coreb.infectiologie.com/UserFiles/File/procedures/rpmo-ethique-rea-covid-19-vf-24-corr26-mar20-2.pdf (accessed March 31, 2020)].

[9] Espace éthique région Ile-de-France. COVID-19 Ethics and community observatory–Ethical decisional considerations in critical care (in French); 2020 [https://www.espace-ethique.org/sites/default/files/reanimation_decision_17.03.20_0.pdf].

[10] Emanuel EJ, Persad G, Upshur R, Thome B, Parker M, Glickman A, et al. Fair Allocation of Scarce Medical Resources in the Time of Covid-19. N Engl J Med 2020. http://dx.doi.org/10.1056/NEJMsb2005114.

[11] Swiss Academy Of Medical Sciences. COVID-19 pandemic: triage for intensive-care treatment under resource scarcity. Swiss Med Wkly 2020;150. http://dx.doi.org/10.4414/smw.2020.20229 [w20229].

[12] White DB, Lo B. A Framework for Rationing Ventilators and Critical Care Beds During the COVID-19 Pandemic. JAMA 2020. http://dx.doi.org/10.1001/jama.2020.5046.

[13] Truog RD, Mitchell C, Daley GQ. The Toughest Triage - Allocating Ventilators in a Pandemic. N Engl J Med 2020. http://dx.doi.org/10.1056/NEJMp2005689.

[14] Henry BM. COVID-19, ECMO, and lymphopenia: a word of caution. Lancet Respir Med 2020. http://dx.doi.org/10.1016/S2213-2600(20)30119-3.

[15] MacLaren G, Fisher D, Brodie D. Preparing for the Most Critically Ill Patients With COVID-19: The Potential Role of Extracorporeal Membrane Oxygenation. JAMA 2020. http://dx.doi.org/10.1001/jama.2020.2342.

[16] Combes A, Cholley B, Lebreton G, Leprince P, Longrois D, Schmidt M, et al. Proposition de plan d'action du groupe de réflexion sur l'usage de l'ECMO pour les patients COVID +; 2020 [https://www.srlf.org/wp-content/uploads/2020/03/RECO-ECMO-APHP-23-03-2020.pdf (accessed March 31, 2020)].

[17] Bhatraju PK, Ghassemieh BJ, Nichols M, Kim R, Jerome KR, Nalla AK, et al. Covid-19 in Critically Ill Patients in the Seattle Region – Case Series. N Engl J Med 2020. http://dx.doi.org/10.1056/NEJMoa2004500.

[18] Christian MD, Hawryluck L, Wax RS, Cook T, Lazar NM, Herridge MS, et al. Development of a triage protocol for critical care during an influenza pandemic. CMAJ Can Med Assoc J J Assoc Medicale Can 2006;175:1377–81. http://dx.doi.org/10.1503/cmaj.060911.

[19] Christian MD, Hamielec C, Lazar NM, Wax RS, Griffith L, Herridge MS, et al. A retrospective cohort pilot study to evaluate a triage tool for use in a pandemic. Crit Care Lond Engl 2009;13:R170. http://dx.doi.org/10.1186/cc8146.

[20] Vincent JL, de Mendonça A, Cantraine F, Moreno R, Takala J, Suter PM, et al. Use of the SOFA score to assess the incidence of organ dysfunction/failure in intensive care units: results of a multicenter, prospective study. Working group on "sepsis-related problems" of the European Society of Intensive Care Medicine. Crit Care Med 1998;26:1793–800.

[21] Zhu N, Zhang D, Wang W, Li X, Yang B, Song J, et al. A Novel Coronavirus from Patients with Pneumonia in China, 2019. N Engl J Med 2020;382:727–33. http://dx.doi.org/10.1056/NEJMoa2001017.

[22] Stevens PE, Levin A, Kidney Disease: Improving Global Outcomes Chronic Kidney Disease Guideline Development Work Group Members. Evaluation and management of chronic kidney disease: synopsis of the kidney disease: improving global outcomes 2012 clinical practice guideline. Ann Intern Med 2013;158:825–30 [10.7326/0003-4819-158-11-201306040-00007].

[23] Christofides C, Moore R, Nel M. Baux Score as a Predictor of Mortality at the CHBAH Adult Burns Unit. J Surg Res 2020;251:53–62. http://dx.doi.org/10.1016/j.jss.2020.01.018.

[24] Rockwood K, Song X, MacKnight C, Bergman H, Hogan DB, McDowell I, et al. A global clinical measure of fitness and frailty in elderly people. CMAJ Can Med Assoc J J Assoc Medicale Can 2005;173:489–95. http://dx.doi.org/10.1503/cmaj.050051.

[25] Guan W, Ni Z, Hu Y, Liang W, Ou C, He J, et al. Clinical Characteristics of Coronavirus Disease 2019 in China. N Engl J Med 2020. http://dx.doi.org/10.1056/NEJMoa2002032 [0:null].

[26] Rosenbaum L. Facing Covid-19 in Italy - Ethics, Logistics, and Therapeutics on the Epidemic's Front Line. N Engl J Med 2020. http://dx.doi.org/10.1056/NEJMp2005492.
