# Supplementary material for: Validation of a pre-established triage protocol for critically ill patients in a COVID-19 outbreak under resource scarcity: A retrospective multicenter cohort study
Source: PLoS One. 2023 May 11;18(5):e0285690. doi: 10.1371/journal.pone.0285690 (PMC10174588; doi:10.1371/journal.pone.0285690)
Supplement: Attachment — Submitted filename: Response to reviewers.docx [file pone.0285690.s009.docx]

Response to reviewers

Dear Editor, Dear reviewers,

We implemented in our manuscript a discussion about the potential benefits of machine learning approach for triage algorithms. The paragraph is called « Triage algorithms and Machine Learning » and is located in the discussion part between the « study limitations » part and the « triage algorithms and ethical guidelines » part.

As requested, we made sure that the manuscript meets PLOS ONE's style requirements. References were also verified.

Best Regards,

Nicolas DONAT
